# Comparison of electrochemical impedance spectra for electrolyte-supported solid oxide fuel cells (SOFCs) and protonic ceramic fuel cells (PCFCs)

**DOI:** 10.1038/s41598-021-90211-9

**Published:** 2021-05-19

**Authors:** Hirofumi Sumi, Hiroyuki Shimada, Yuki Yamaguchi, Yasunobu Mizutani, Yuji Okuyama, Koji Amezawa

**Affiliations:** 1 Innovative Functional Materials Research Institute, National Institute of Advanced Industrial Science and Technology (AIST), Nagoya, Aichi 463-8560 Japan; 2 Faculty of Engineering, University of Miyazaki, Miyazaki, 889-2192 Japan; 3 Institute of Multidisciplinary Research for Advanced Materials, Tohoku University, Sendai, Miyagi 980-8577 Japan

**Keywords:** Fuel cells, Fuel cells

## Abstract

Protonic ceramic fuel cells (PCFCs) are expected to achieve high power generation efficiency at intermediate temperature around 400–600 °C. In the present work, the distribution of relaxation times (DRT) analysis was investigated in order to deconvolute the anode and cathode polarization resistances for PCFCs supported on yttria-doped barium cerate (BCY) electrolyte in comparison with solid oxide fuel cells (SOFCs) supported on scandia-stabilized zirconia (ScSZ) electrolyte. Four DRT peaks were detected from the impedance spectra measured at 700 °C excluding the gas diffusion process for ScSZ and BCY. The DRT peaks at 5 × 10^2^–1 × 10^4^ Hz and 1 × 10^0^–2 × 10^2^ Hz were related to the hydrogen oxidation reaction at the anode and the oxygen reduction reaction at the cathode, respectively, for both cells. The DRT peak at 2 × 10^1^–1 × 10^3^ Hz depended on the hydrogen concentration at the anode for ScSZ, while it was dependent on the oxygen concentration at the cathode for BCY. Compared to ScSZ, steam was produced at the opposite electrode in the case of BCY, which enhanced the cathode polarization resistance for PCFCs.

## Introduction

Solid oxide fuel cells (SOFCs) are expected to be power generation systems with high energy conversion efficiency. The power generation efficiency of commercial stationary SOFC systems using yttria- or scandia-stabilized zirconia (YSZ or ScSZ) electrolyte manufactured by Kyocera–Aisin (0.7 kW) and Bloom Energy (200 kW) has reached to 53–65% (LHV; lower heating value). The electrode reactions of SOFCs are as follows:

1 $${\text{Anode: 2H}}_{{2}} + {\text{2O}}^{{{2} - }} \to {\text{2H}}_{{2}} {\text{O}} + {\text{4e}}^{ - }$$

2 $${\text{Cathode: O}}_{{2}} + {\text{4e}}^{ - } \to {\text{2O}}^{{{2} - }}$$

Owing to the supply of oxide ions (O^2−^) from a cathode to an anode through an electrolyte at high temperatures above 700 °C, SOFCs can, in principle, directly use hydrocarbons and alcohol fuels as well as hydrogen^1–5^. Steam is produced at the anode by power generation, which promotes the steam reforming of hydrocarbons and alcohol fuels. The power generation efficiency is proportional to the output voltage and fuel utilization. However, increasing the fuel utilization is limited in the case of SOFCs, because the fuel is diluted at the outlet by the steam production at the anode.

Iwahara et al.^6,7^ discovered the proton (H^+^) conducting ceramics of perovskite-type *AB*_1−*x*_*M*_*x*_O_3−δ_ (*A* = Ba, Sr, Ca; *B* = Ce, Zr; *M* = Y, In, Nd, Yb). The electrode reactions of protonic ceramic fuel cells (PCFCs) are as follows:

3 $${\text{Anode: H}}_{{2}} \to {\text{2H}}^{ + } + {\text{2e}}^{ - }$$

4 $${\text{Cathode: }}\raise.5ex\hbox{$\scriptstyle 1$}\kern-.1em/ \kern-.15em\lower.25ex\hbox{$\scriptstyle 2$} {\text{O}}_{{2}} + {\text{2H}}^{ + } + {\text{2e}}^{ - } \to {\text{H}}_{{2}} {\text{O}}$$

In this case, the fuel is not diluted by power generation, because steam is produced at the cathode. PCFCs are expected to realize high fuel utilization compared to SOFCs^8^. Furthermore, the ionic conductivities of yttria-doped barium cerate (BCY) and zirconate (BZY) are higher than that of YSZ at intermediate temperatures around 400–600 °C^9^. The maximum power densities were 0.6–1.3 W/cm^2^ at 600 °C for anode-supported PCFCs using BCY/BZY-based electrolytes^10–12^.

Dissolving transition metal elements such as manganese, iron, cobalt and nickel into BCY^13^ and BZY^14^ decreases the ionic conductivity. While nickel dissolving into BCY and BZY acted as a sintering aid during the co-sintering of the electrolyte thin-film and anode substrate for anode-supported cells during cell manufacturing^15–17^, it deteriorated the ionic conductivity and proton transport number^18–20^. Transition metal elements also possibly diffuse from cathode materials. Therefore, the deconvolution technique of the anode and cathode polarization resistances is required for evaluating the initial performance and durability of PCFCs.

The polarization resistances were frequently evaluated by the electrochemical impedance spectroscopy (EIS) for various devices such as fuel cells and batteries. An equivalent circuit model is required for complex nonlinear least square (CNLS) fitting to separate each resistance. However, it is sometimes difficult to assume the equivalent circuit model with appropriate initial parameters for the CNLS fitting. The distribution of relaxation times (DRT) analysis is a powerful tool to deconvolute EIS^21–23^, which are widely used for fuel cells^24–28^, electrolysis cells^29^, lithium-ion batteries^30^ and supercapacitors^31^. For SOFCs^24–26^ and polymer electrolyte fuel cells (PEFCs)^27^, the anode and cathode polarization resistances were separated with high resolution. The ratios of the anode to cathode polarization resistances were 75:25 and 4:96 for SOFCs^28^ and PEFCs^27^, respectively, which was caused by the difference in electrode reactions as shown in Eqs. (1, 2) and (3, 4). DRT analysis is also a powerful tool for investigating the degradation mechanism during durability tests^32^.

In the present work, the deconvolution technique of the anode and cathode polarization resistances was investigated for PCFCs. While some researchers have applied the DRT analysis to anode-supported PCFCs^33,34^, the physicochemical origins of each DRT peak should be more discussed. We prepared electrolyte-supported SOFCs and PCFCs in order to attach a reference electrode. The ohmic resistances of electrolyte-supported cells are large due to thick electrolyte compared to anode-supported cells. ScSZ and BCY are selected as electrolytes for SOFCs and PCFCs, respectively, because these conductivities are higher than those of YSZ^35^ and BZY^36^. The difference in elementary electrode reactions was discussed using the results of DRT analysis for SOFCs and PCFCs.

## Results

**Figure 1:**
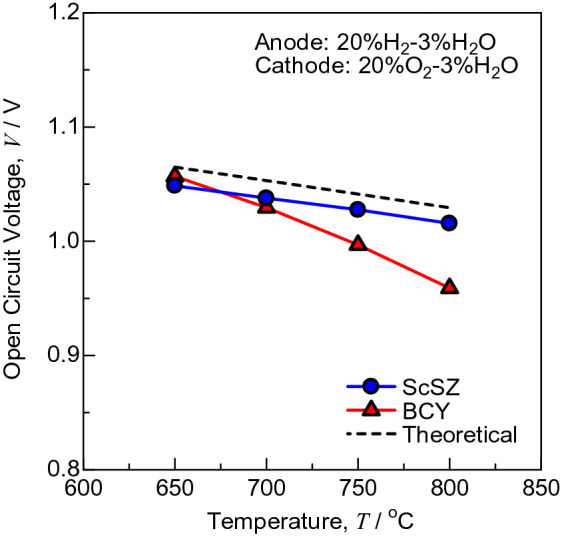
Temperature dependence of OCVs in H_2_:H_2_O:N_2_ = 20:3:77 vol.% (anode) and O_2_:H_2_O:N_2_ = 20:3:77 vol.% (cathode) for SOFCs supported on ScSZ electrolyte and PCFCs supported on BCY electrolyte. The dotted line indicates the theoretical EMF, when the transport number is assumed to be unity.

The theoretical electromotive forces (EMFs; *V*_th_) are as shown in Eqs. (5) and (6) for SOFCs and PCFCs, respectively:

5 $$V_{{t{\text{h}}}} = t_{{{\text{O}}^{2 - } }} \frac{RT}{{4F}}\ln \frac{{p_{{{\text{O}}_{{2}} {\text{,c}}}} }}{{p_{{{\text{O}}_{{2}} {\text{,a}}}} }} = t_{{{\text{O}}^{2 - } }} \frac{RT}{{4F}}\ln \frac{{p_{{{\text{O}}_{{2}} {\text{,c}}}} Kp_{{{\text{H}}_{{2}} {\text{,a}}}}^{2} }}{{p_{{{\text{H}}_{{2}} {\text{O,a}}}}^{2} }}$$

6 $$V_{{t{\text{h}}}} = - t_{{{\text{H}}^{ + } }} \frac{RT}{{2F}}\ln \frac{{p_{{{\text{H}}_{{2}} {\text{,c}}}} }}{{p_{{{\text{H}}_{{2}} {\text{,a}}}} }} = - t_{{{\text{H}}^{ + } }} \frac{RT}{{2F}}\ln \frac{{p_{{{\text{H}}_{{2}} {\text{O,c}}}} }}{{p_{{{\text{H}}_{{2}} {\text{,a}}}} K^{1/2} p_{{{\text{O}}_{{2}} {\text{,c}}}}^{1/2} }}$$

where *R* is the gas constant, *T* is the absolute temperature, *F* is the Faraday constant, *p*_a_ and *p*_c_ are the partial pressures at the anode and cathode, respectively, and *K* is the equilibrium constant of 2H_2_(g) + O_2_(g) → 2H_2_O(g). The *t* is the transport number, which is dependent on $p_{{{\text{H}}_{{2}} }} ,p_{{{\text{H}}_{{2}} {\text{O}}}} \;{\text{and}}\;p_{{{\text{O}}_{{2}} }}$ for some electrolytes. Hole conduction appears for proton-conductive ceramics such as BCY and BZY at high temperature and oxygen partial pressure^37^, which leads to a decrease in power generation efficiency^38^. Figure 1 shows the temperature dependence of OCVs in H_2_:H_2_O:N_2_ = 20:3:77 vol.% (anode) and O_2_:H_2_O:N_2_ = 20:3:77 vol.% (cathode) for SOFCs supported on ScSZ electrolyte and PCFCs supported on BCY electrolyte. The dotted line indicates the theoretical EMF, when the transport number is assumed to be unity. The theoretical EMFs of ScSZ and BCY are almost the same in these atmospheres. The OCVs were 15 mV lower than the theoretical EMFs for ScSZ due to gas leakage from the sealant. While the slope of the OCVs was nearly matched that of the theoretical EMFs for ScSZ, it was quite different for BCY. The OCVs were 45 and 70 mV lower than the theoretical EMFs for BCY at 750 and 800 °C, respectively, owing to hole conduction. ScSZ and BCY exhibited nearly the same OCVs at ≤ 700 °C, suggesting the prevention of hole conduction in BCY. The transport number of holes for BCY-based electrolyte is smaller than that for BZY, while the transport number of oxide ions for BCY-based electrolyte is slightly larger than that for BZY^39^. The impedance was measured precisely at 700 °C in the present work.

**Figure 2:**
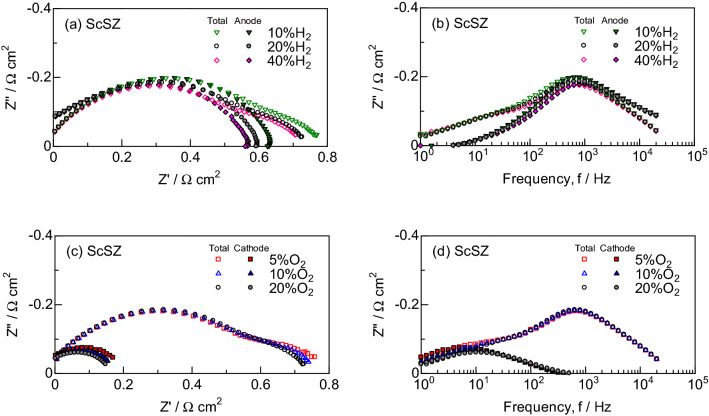
IR-free impedance spectra at 700 °C in (**a**, **b**) the constant oxidant of O_2_:H_2_O:N_2_ = 20:3:77 vol.% and (**c**, **d**) the constant fuel of H_2_:H_2_O:N_2_ = 20:3:77 vol.% for SOFCs supported on ScSZ electrolyte.

**Figure 3:**
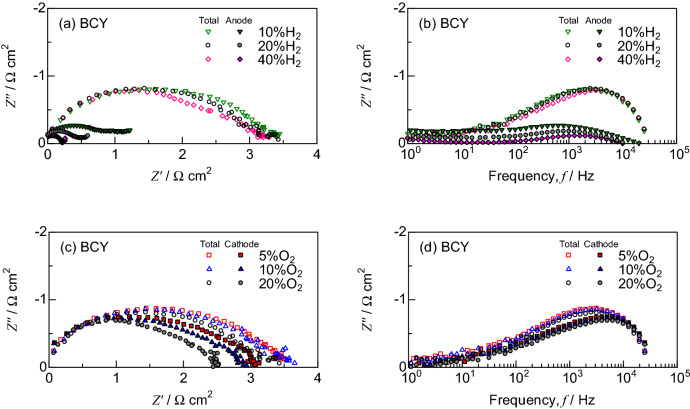
IR-free impedance spectra at 700 °C in (**a**, **b**) the constant oxidant of O_2_:H_2_O:N_2_ = 20:3:77 vol.% and (**c**, **d**) the constant fuel of H_2_:H_2_O:N_2_ = 20:3:77 vol.% for PCFCs supported on BCY electrolyte.

Figures 2 and 3 show the IR-free impedance spectra at 700 °C for SOFCs supported on ScSZ electrolyte and PCFCs supported on BCY electrolyte. The ohmic resistances were subtracted to compare the polarization resistances among anode–cathode (total), anode-reference electrode and cathode-reference electrode impedances in these figures. The polarization resistance of the anode (Fig. 2a) was larger than that of the cathode (Fig. 2c) for ScSZ, while that of the anode (Fig. 3a) was smaller than that of the cathode (Fig. 3c) for BCY. When the hydrogen concentration was changed from 10 to 40 vol.% in the constant oxidant composition of O_2_:H_2_O:N_2_ = 20:3:77 vol.%, the impedance around 1 kHz decreased for ScSZ (Fig. 2b) and BCY (Fig. 3b). On the other hand, the impedance around 10 Hz decreased by the change from 5 to 20 vol.% O_2_ in the constant fuel composition of H_2_:H_2_O:N_2_ = 20:3:77 vol.% for ScSZ (Fig. 2d), while the impedance decreased across a wide range frequency for BCY (Fig. 3d).

**Figure 4:**
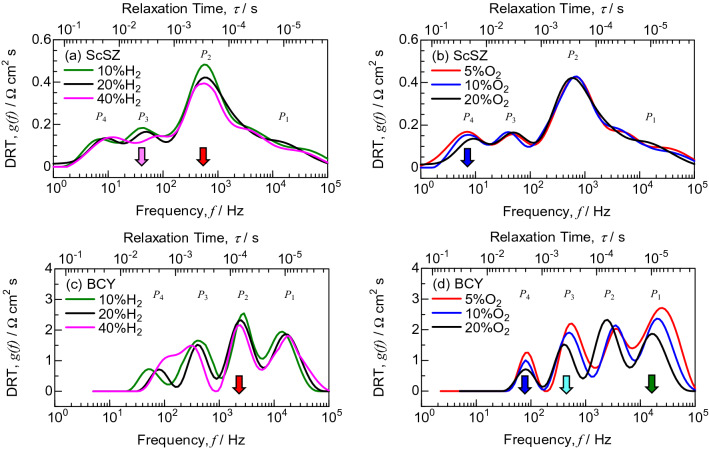
DRT spectra at 700 °C in (**a**, **c**) the constant oxidant of O_2_:H_2_O:N_2_ = 20:3:77 vol.% and (**b**, **d**) the constant fuel of H_2_:H_2_O:N_2_ = 20:3:77 vol.% for SOFCs supported on ScSZ electrolyte and PCFCs supported on BCY electrolyte.

The anode-reference electrode and cathode-reference electrode impedances gave rough information about anode and cathode polarization resistances, because the electrochemical potentials of reference electrodes were sensitive against ac amplitude during impedance measurements^40,41^. In the present work, the DRT analysis suggests the deconvolution of the anode and cathode polarization resistances using anode–cathode impedance as shown in Fig. 4, and the DRT peaks were assigned with reference to the dependence of hydrogen and oxygen concentrations in the anode and cathode, respectively. Unfortunately, the impedance spectra were noisy below 10 Hz, because gas leakage might slightly occur through the grain boundary of the BCY electrolyte with insufficient sintering. Ivers-Tiffée and Weber^28^ reported the effect of experimental errors on the DRT analysis. The resolution of EIS deconvolution decreases for data with errors. However, the results of Fig. 4 are acceptable, because the gaps of each DRT peak are approximately one order of magnitude against frequency. The DRT analysis was performed using impedance data at frequencies ≥ 10 Hz for BCY. Four DRT peaks were detected for ScSZ and BCY. Although the DRT peak for the gas diffusion process is generally observed below 10 Hz, it is negligible for the electrolyte-supported cells with the thin anode and cathode in the present work. For ScSZ, the *P*_2_ and *P*_3_ peaks decreased with increasing hydrogen concentration at the anode (Fig. 4a), while the *P*_4_ peak decreased with increasing oxygen concentration at the cathode (Fig. 4b). The change in the *P*_1_ peak was small for ScSZ. For BCY, the *P*_2_ peak decreased with increasing hydrogen concentration at the anode (Fig. 4c), while the *P*_1_, *P*_3_ and *P*_4_ peaks decreased with increasing oxygen concentration at the cathode (Fig. 4d). The dependence of the *P*_3_ peak was different between ScSZ and BCY.

It was previously reported that the DRT peak around 10–100 kHz could be ascribed to the ionic conduction process in mixed ionic-electronic conductors^26,33^. This polarization resistance is dependent on the contact condition and the microstructure of the cathode. The *P*_1_ peak for BCY was relatively larger than that for ScSZ owing to insufficient contact between LSCF and BCY in the present work. The *P*_2_ and *P*_4_ peaks are dependent on the hydrogen concentration at the anode and the oxygen concentration at the cathode, respectively, for both cells. The *P*_2_ peak is related to the hydrogen oxidation reaction, while the *P*_4_ peak is related to the oxygen reduction reaction. On the other hand, the *P*_3_ peak is dependent on the hydrogen concentration at the anode for ScSZ, and the oxygen concentration at the cathode for BCY. Compared to SOFCs, steam was produced at the opposite electrode in the case of PCFCs as shown in Eqs. (1)–(4). Therefore, the *P*_3_ peak is related to the steam production reaction.

The elementary electrode reactions for SOFCs and PCFCs are as follows.

### Gas diffusion

7 $${\text{Anode gas diffusion: H}}_{{2}} \left( {\text{g}} \right) \to {\text{H}}_{{2}} \left( {\text{g}} \right)$$

8 $${\text{Cathode gas diffusion: O}}_{{2}} \left( {\text{g}} \right) \to {\text{O}}_{{2}} \left( {\text{g}} \right)$$

9 $${\text{Steam diffusion: 2H}}_{{2}} {\text{O}}\left( {\text{g}} \right) \to {\text{2H}}_{{2}} {\text{O}}\left( {\text{g}} \right)$$

### Ionic diffusion

10 $${\text{Bulk and interface diffusion: 2O}}^{{{2} - }} \to {\text{2O}}^{{{2} - }}$$

### Hydrogen oxidation reaction

11 $${\text{Dissociative adsorption: 2H}}_{{2}} \left( {\text{g}} \right) \to {\text{4H}}_{{{\text{ad}},{\text{surf}}}}$$

12 $${\text{Surface diffusion: 4H}}_{{{\text{ad}},{\text{surf}}}} \to {\text{4H}}_{{{\text{ad}},{\text{TPB}}}}$$

13 $${\text{Charge transfer: 4H}}_{{{\text{ad}},{\text{TPB}}}} \to {\text{4H}}^{ + } + {\text{4e}}^{ - }$$

### Oxygen reduction reaction

14 $${\text{Dissociative adsorption: O}}_{{2}} \left( {\text{g}} \right) \to {\text{2O}}_{{{\text{ad}},{\text{surf}}}}$$

15 $${\text{Charge transfer: 2O}}_{{{\text{ad}},{\text{surf}}}} + {\text{4e}}^{ - } \to {\text{2O}}^{{{2} - }}$$

### Steam production

16 $${\text{Steam formation: 4H}}^{ + } + {\text{2O}}^{{{2} - }} \to {\text{2H}}_{{2}} {\text{O}}_{{{\text{ad}},{\text{TPB}}}}$$

17 $${\text{Steam desorption: 2H}}_{{2}} {\text{O}}_{{{\text{ad}},{\text{TPB}}}} \to {\text{2H}}_{{2}} {\text{O}}\left( {\text{g}} \right)$$

where the subscripts “ad,surf” and “ad,TPB” represent the adsorbed atom at the surface and triple phase boundary, respectively. Intermediate products such as O^−^ and OH^−^ are temporarily formed during charge transfer (Eqs. (13) and (15)).

**Figure 5:**
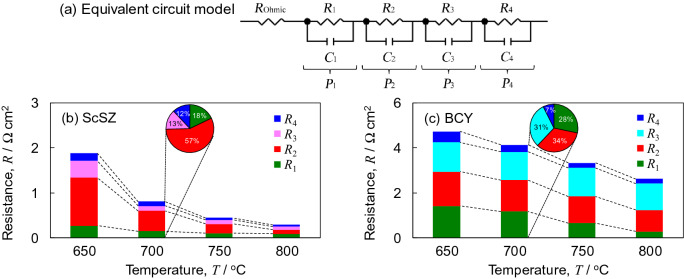
(**a**) Equivalent circuit model with a series connection of resistance (*R*) and four parallel resistance–capacitance (*RC*) elements. (**b**, **c**) Temperature dependence of polarization resistances in H_2_:H_2_O:N_2_ = 20:3:77 vol.% (anode) and O_2_:H_2_O:N_2_ = 20:3:77 vol.% (cathode) for SOFCs supported on ScSZ electrolyte and PCFCs supported on BCY electrolyte. The pie chart depict the ratios of the polarization resistance at 700 °C.

The polarization resistances are derived using the equivalent circuit model of Fig. 5a with reference to the result of DRT analysis. Figures 5b,c show the temperature dependence of the polarization resistances in H_2_:H_2_O:N_2_ = 20:3:77 vol.% (anode) and O_2_:H_2_O:N_2_ = 20:3:77 vol.% (cathode) for SOFCs supported on ScSZ electrolyte and PCFCs supported on BCY electrolyte. All polarization resistances for BCY was larger than those for ScSZ. The contact condition between the electrolyte and cathode should be modified to decrease *R*_1_. The nickel dissolved into BCY in the composite anode and at the interface between the anode and electrolyte, which enhanced *R*_2_ for the hydrogen oxidation reaction^19^. On the other hand, LSCF is well-known as a mixed oxide ionic-electronic conductor. However, proton is not conducted in LSCF. *R*_3_ was enhanced for PCFCs, because the active area for steam production is limited to the two-dimensional interface between the BCY electrolyte and LSCF cathode. *R*_4_ for oxygen reduction reaction for BCY was three times as large as that for ScSZ. The difference in *R*_4_ was smaller than those of the other polarization resistances, as the oxygen reduction reactions of Eqs. (14) and (15) are the same for BCY and ScSZ. The pie charts in Fig. 5b,c depict the ratios of the polarization resistance at 700 °C. The ratios of the anode to cathode polarization resistances were 70:30 and 34:66 for ScSZ and BCY, respectively. For BCY, the DRT peaks were slightly affected by the conductivity of oxide ions. Therefore, we are going to perform DRT analysis for the other electrolytes with proton conductivity such as BZY in order to more investigate the assignment of DRT peaks to each elementary reaction process in the near future.

## Conclusion

In the present work, the deconvolution technique of the anode and cathode polarization resistances was investigated using DRT analysis for PCFCs supported on a BCY electrolyte in comparison with SOFCs supported on an ScSZ electrolyte. The OCVs for BCY were nearly equal to those for ScSZ and theoretical EMFs at ≤ 700 °C, suggesting the prevention of hole conduction. Four DRT peaks were detected from the impedance spectra measured at 700 °C for ScSZ and BCY. The DRT peak for the gas diffusion process was negligible for electrolyte-supported cells. The DRT peak at 1 × 10^4^–5 × 10^4^ Hz, *P*_1_, was ascribed to the bulk diffusion of oxide ions in the mixed ionic-electronic conductor of LSCF and interface diffusion between the cathode and electrolyte. The DRT peaks at 5 × 10^2^–1 × 10^4^ Hz, *P*_2_, and 1 × 10^0^–2 × 10^2^ Hz, *P*_4_, were related to the hydrogen oxidation reaction at the anode and the oxygen reduction reaction at the cathode, respectively. On the other hand, the DRT peak at 2 × 10^1^–1 × 10^3^ Hz, *P*_3_, was dependent on the hydrogen concentration at the anode for ScSZ, and on the oxygen concentration at the cathode for BCY owing to steam production at the opposite electrodes in SOFCs and PCFCs. The ratios of anode to cathode polarization resistances were 70:30 and 34:66 at 700 °C for ScSZ and BCY, respectively. The polarization resistances of the electrode on the side where steam was produced increased for SOFCs and PCFCs.

## Methods

### Cell preparation

**Figure 6:**
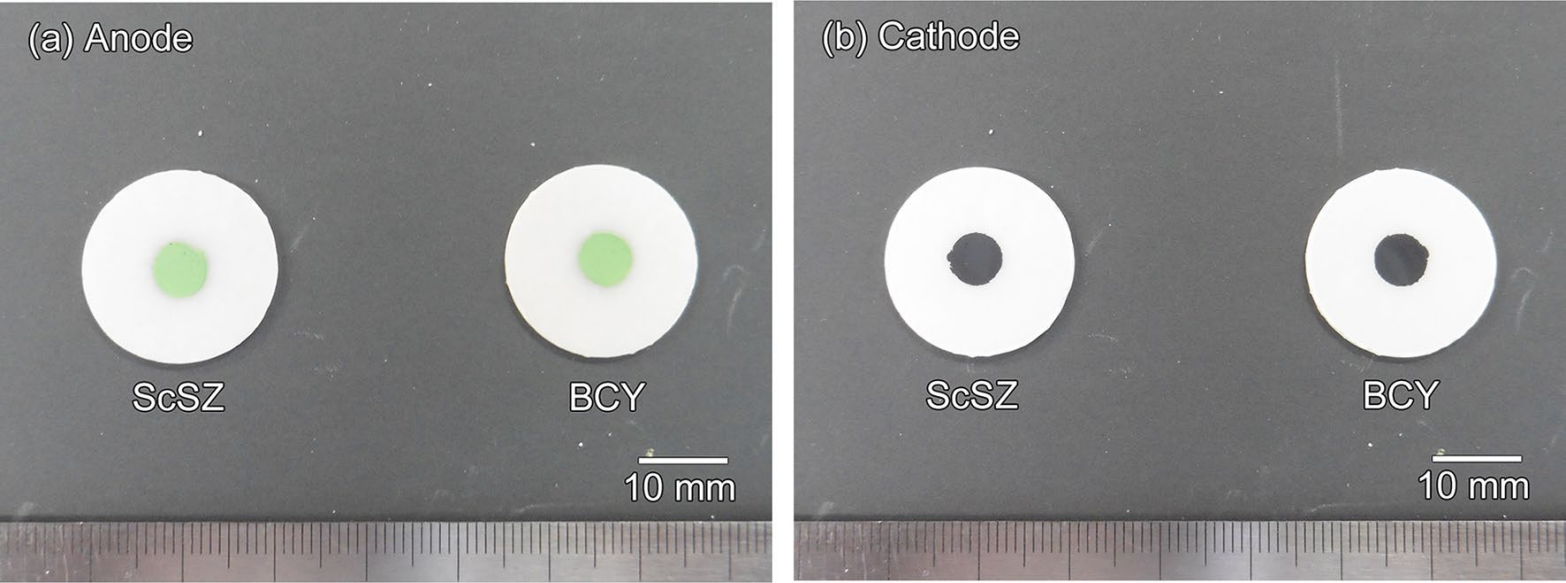
(**a**) Anode and (**b**) cathode sides of SOFCs supported on ScSZ electrolyte and PCFCs supported on BCY electrolyte.

Table 1 shows the cell configurations of SOFCs supported on ScSZ electrolyte and PCFCs supported on BCY electrolyte. Commercial powders of (Sc_2_O_3_)_0.10_(CeO_2_)_0.01_(ZrO_2_)_0.89_ (ScSZ; Daiichi Kigenso), BaCe_0.8_Y_0.2_O_3−δ_ (BCY; Kusaka Rare Metal), Ce_0.8_Gd_0.2_O_1.95_ (GDC; Kusaka Rare Metal), NiO (Sumitomo metal mining) and La_0.6_Sr_0.4_Co_0.2_Fe_0.8_O_3−δ_ (LSCF; Kusaka Rare Metal) were used as raw materials. The ScSZ and BCY powders were pelletized to 30 mm diameter under a uniaxial pressure of 50 MPa, and then cold-isostatic pressed at 300 MPa. The ScSZ and BCY pellets were sintered in air for 10 h at 1500 and 1550 °C, respectively. The diameter and thickness of the electrolyte pellets were 23 and 1.0 mm, respectively, after sintering. A GDC slurry was prepared by mixing GDC powder, polyvinyl butyral binder (Sekisui Chemical) tallow propylene diamine dispersant (Kao) and dioctyl adipate plasticizer (Wako Pure Chemical) into ethanol and toluene solvents. The GDC interlayer was formed by spin-coating on one side of the ScSZ electrolyte for preventing chemical reactions between ScSZ and LSCF, while no GDC interlayer was formed on the BCY electrolyte. Ni-ScSZ, Ni-BCY (50:50 vol.%) and LSCF pastes were prepared by mixing the raw powders, α-terpineol (Kanto Chemical) and ethyl cellulose (45 cp; Kishida Chemical) using a planetary centrifugal mixer (Thinky ARE-310). The Ni-ScSZ and Ni-BCY anode pastes were screen-printed onto the ScSZ and BCY electrolytes, respectively, and then sintered in air for 2 h at 1300 °C. The LSCF cathode paste was screen-printed on the opposite side of the GDC interlayer and BCY electrolyte, and then sintered in air for 1 h at 1050 °C. The SOFCs supported on the ScSZ electrolyte and PCFCs supported on the BCY electrolyte are shown in Fig. 6.

**Table 1:** Cell configurations of SOFCs supported on ScSZ electrolyte and PCFCs supported on BCY electrolyte.

Electrolyte	Anode	Interlayer	Cathode
ScSZ	Ni-ScSZ	GDC	LSCF
BCY	Ni-BCY	None	LSCF

### Electrochemical evaluation

The electrochemical properties were measured using a potentiostat/galvanostat (Solartron Analytical 1470E) and an impedance analyzer (Solartron Analytical 1455). Platinum meshes were used as current collectors. A reference electrode of platinum wire was fixed around the ScSZ and BCY pellets using platinum paste. A mixture of H_2_:H_2_O:N_2_ = 20:3:77 vol.% and O_2_:H_2_O:N_2_ = 20:3:77 vol.% were supplied as fuel and oxidant, respectively, at a flow rate of 100 mL/min. The cells were heated up to 800 °C, followed by the reduction treatment of Ni-based anodes and hydration treatment of the BCY electrolyte for 24 h. The AC impedance was measured between the anode–cathode, anode-reference electrode and cathode-reference electrode under open circuit voltage (OCV) from 100 kHz to 0.1 Hz with 20 steps per logarithmic decade in H_2_:H_2_O:N_2_ = *x*:3:(97 − *x*) vol.% (*x* = 10, 20, 40) and O_2_:H_2_O:N_2_ = *y*:3:(97 − *y*) vol.% (*y* = 5, 10, 20) at 800, 750, 700 and 650 °C.

The distribution of relaxation time (DRT) analysis was performed using Z-Assist software (Toyo Corp.)^32^. The real impedance was used for DRT analysis owing to the reduced effects of measurement errors and inductive components compared to the imaginary parts^28^. After DRT analysis, the parameters were refined by CNLS fitting using ZView software (Scribner Associates) assuming an equivalent circuit model with a series connection of resistance (*R*) and four parallel resistance–capacitance (*RC*) elements.

## Data Availability

The data that support the findings of this study are available from the corresponding author upon reasonable request.

## References

[1] Eguchi K, Kojo H, Takeguchi T, Kikuchi R, Sasaki K (2002). Fuel flexibility in power generation by solid oxide fuel cells. Solid State Ionics.

[2] Murray EP, Tsai T, Barnett SA (1999). A direct-methane fuel cell with a ceria-based anode. Nature.

[3] Park SD, Vohs JM, Gorte RJ (2000). Direct oxidation of hydrocarbons in a solid-oxide fuel cell. Nature.

[4] Sumi H, Ukai K, Mizutani Y, Mori H, Wen C-J, Takahashi H, Yamamoto O (2004). Performance of nickel-scandia-stabilized zirconia cermet anodes for SOFCs in 3%H_2_O–CH_4_. Solid State Ionics.

[5] Sumi H, Yamaguchi T, Hamamoto K, Suzuki T, Fujishiro Y (2012). Impact of direct butane microtubular solid oxide fuel cells. J. Power Sources.

[6] Iwahara H, Esaka T, Uchida H, Maeda N (1981). Proton conduction in sintered oxides and its application to steam electrolysis for hydrogen production. Solid State Ionics.

[7] Iwahara H (1995). Technological challenges in the application of proton conducting ceramics. Solid State Ionics.

[8] Matsuzaki Y, Tachikawa Y, Somekawa T, Hatae T, Matsumoto H, Taniguchi S, Sasaki K (2015). Effect of proton-conduction in electrolyte on electric efficiency of multi-stage solid oxide fuel cells. Sci. Rep..

[9] Fabbri E, Pergolesi D, Traversa E (2010). Electrode materials: A challenge for the exploitation of protonic solid oxide fuel cells. Sci. Technol. Adv. Mater..

[10] Duan C, Tong J, Shang M, Nikodemski S, Sanders M, Ricote S, Almansoori A, O’hayer R (2015). Readily processed protonic ceramic fuel cells with high performance at low temperatures. Science.

[11] Choi S, Kucharczyk CJ, Liang Y, Zhang X, Takeuchi I, Ji H-I, Haile SM (2018). Exceptional power density and stability at intermediate temperatures in protonic ceramic fuel cells. Nat. Energy.

[12] An H, Lee H-W, Kim B-K, Son J-W, Yoon KJ, Kim H, Shin D, Ji H-I, Lee J-H (2018). A 5 × 5 cm^2^ protonic ceramic fuel cell with a power density of 1.3 W cm^−^^2^ at 600 °C. Nat. Energy.

[13] Shimura T, Tanaka H, Matsumoto H, Yogo T (2005). Influence of the transition-metal doping on conductivity of a BaCeO_3_-based protonic conductor. Solid State Ionics.

[14] Han D, Otani Y, Goto K, Uemura S, Majima M, Uda T (2020). Electrochemical and structural influence on BaZr_0.8_Y_0.2_O_3__−__*δ*_ from manganese, cobalt, and iron oxide additives. J. Am. Ceram. Soc..

[15] Yamaguchi T, Shimada H, Honda U, Kishimoto H, Ishiyama T, Hamamoto K, Sumi H, Suzuki T, Fujishiro Y (2016). Development of anode-supported electrochemical cell based on proton-conductive Ba(Ce, Zr)O<sub>3</sub> electrolyte. Solid State Ionics.

[16] Shimada H, Yamaguchi T, Sumi H, Yamaguchi Y, Nomura K, Fujishiro Y (2018). Effect of Ni diffusion into BaZr_0.1_Ce_0.7_Y_0.1_Yb_0.1_O_3__−__*δ*_ electrolyte during high temperature co-sintering in anode-supported solid oxide fuel cells. Ceram. Int..

[17] Liu Z, Wang X, Liu M, Liu J (2018). Enhancing sinterability and electrochemical properties of Ba(Zr_0.1_Ce_0.7_Y_0.2_)O_3__−__*δ*_ proton conducting electrolyte for solid oxide fuel cells by addition of NiO. Int. J. Hydrogen Energy.

[18] Han D, Shinoda K, Tsukimoto S, Takeuchi H, Hiraiwa C, Majima M, Uda T (2014). Origins of structural and electrochemical influence on Y-doped BaZrO_3_ heat-treated with NiO additive. J. Mater. Chem. A.

[19] Onishi T, Han D, Noda Y, Hatada N, Majima M, Uda T (2018). Evaluation of performance and durability of Ni–BZY cermet electrodes with BZY electrolyte. Solid State Ionics.

[20] Han D, Uemura S, Hiraiwa C, Majima M, Uda T (2018). Detrimental effect of sintering additives on conducting ceramics: Yttrium-doped barium zirconate. Chemsuschem.

[21] Schichlein H, Muller AC, Voigts M, Krugel A, Ivers-Tiffée E (2002). Deconvolution of electrochemical impedance spectra for the identification of electrode reaction mechanisms in solid oxide fuel cells. J. Appl. Electrochem..

[22] Boukamp BA, Rolle A (2017). Analysis and application on distribution of relaxation times in solid state ionics. Solid State Ionics.

[23] Wan TH, Saccoccio M, Chen C, Ciucci F (2015). Influence of the discretization methods on the distribution of relaxation times deconvolution: Implementing radial basis functions with DRTtools. Solid State Ionics.

[24] Leonide A, Rüger B, Weber A, Meulenberg WA, Ivers-Tiffée E (2010). Evaluation and modeling of the cell resistance in anode-supported solid oxide fuel cells. J. Electrochem. Soc..

[25] Sumi H, Yamaguchi T, Hamamoto K, Suzuki T, Fujishiro Y, Matsui T, Eguchi K (2012). AC impedance characteristics for anode-supported microtubular solid oxide fuel cells. Electrochim. Acta.

[26] Sumi H, Yamaguchi T, Hamamoto K, Suzuki T, Fujishiro Y (2013). High performance of La_0.6_Sr_0.4_Co_0.2_Fe_0.8_O_3_–Ce_0.9_Gd_0.1_O_1.95_ nanoparticulate cathode for intermediate temperature microtubular solid oxide fuel cells. J. Power Sources.

[27] Heizmann M, Weber A, Ivers-Tiffée E (2018). Advanced impedance study of polymer electrolyte membrane single cells by means of distribution of relaxation times. J. Power Sources.

[28] Ivers-Tiffée E, Weber A (2017). Evaluation of electrochemical impedance spectra by the distribution of relaxation times. J. Ceram. Soc. Jpn..

[29] Craves C, Ebbesen SD, Mogensen M (2011). Co-electrolysis of CO_2_ and H_2_O in solid oxide cells: Performance and durability. Solid State Ionics.

[30] Schmidt JP, Chrobak T, Ender M, Illig J, Klotz D, Ivers-Tiffée E (2011). Studies on LiFePO_4_ as cathode material using impedance spectroscopy. J. Power Sources.

[31] Oz A, Hershkovitz S, Belman N, Tal-Gutelmacher E, Tsur Y (2016). Analysis of impedance spectroscopy of aqueous supercapacitors by evolutionary programming: Finding DFRT from complex capacitance. Solid State Ionics.

[32] Sumi H, Shimada H, Yamaguchi Y, Yamaguchi T, Fujishiro Y (2020). Degradation evaluation by distribution of relaxation times analysis for microtubular solid oxide fuel cells. Electrochim. Acta.

[33] Lim D-K, Kim J-H, Chavan AU, Lee T-R, Yoo YS, Song S-J (2016). Performance of proton-conducting ceramic-electrolyte fuel cell with BZCY40 electrolyte and BSCF5582 cathode. Ceram. Int..

[34] Shi N, Su F, Huan D, Xie Y, Lin J, Tan W, Peng R, Xia C, Chen C, Lu Y (2017). Performance and DRT analysis of P-SOFCs fabricated using new phase inversion combined tape casting technology. J. Mater. Chem. A.

[35] Inaba H, Tagawa H (1996). Ceria-based solid electrolytes. Solid State Ionics.

[36] Fabbri E, D'Epifanio A, Di Bartolomeo E, Licoccia S, Traversa E (2008). Tailoring the chemical stability of Ba(Ce<sub>0.8</sub><sub>−</sub><sub>x</sub>Zr<sub>x</sub>)Y<sub>0.2</sub>O<sub>3</sub><sub>−</sub><sub>δ</sub> protonic conductors for intermediate temperature solid oxide fuel cells (IT-SOFCs). Solid State Ionics.

[37] Nomura K, Kageyama H (2007). Transport properties of Ba(Zr<sub>0.8</sub>Y<sub>0.2</sub>)O<sub>3</sub><sub>−</sub><sub>δ</sub> perovskite. Solid State Ionics.

[38] Nakamura T, Mizunuma S, Kimura Y, Mikami Y, Yamauchi K, Kuroha T, Taniguchi N, Tsuji Y, Okuyama Y, Amezawa K (2018). Energy efficiency of ionic transport through proton conducting ceramic electrolytes for energy conversion applications. J. Mater. Chem. A.

[39] Duan C, Kee R, Zhu H, Sullivan N, Zhu L, Bian L, Jennings D, O’Hayre R (2019). Highly efficient reversible protonic ceramic electrochemical cells for power generation and fuel production. Nat. Energy.

[40] Winkler J, Hendriksen PV, Bonanos N, Mogensen M (1998). Geometric requirements of solid electrolyte cells with a reference electrode. J. Electrochem. Soc..

[41] Adler SB, Henderson BT, Wilson MA, Taylor DM, Richards RE (2000). Reference electrode placement and seals in electrochemical oxygen generators. Solid State Ionics.

